# Experiments with Bichloride of Mercury

**Published:** 1893-09

**Authors:** Carrie M. Stewart


### THE DENTAL REGISTER.

VOL. XLVII.] SEPTEMBER, 1893. [NO. 9.

### COMMUNICATIONS.

### Experiments with Bichloride of Mercury.

BY CARRIE M. STEWART, D.D.S., JUNE, 1893.

Because of the peculiar action of bichloride of mercury upon material of an albuminous nature its efficacy as a germicide is believed, by some scientists, to be less than the standard usually assigned to it.

When brought in contact with the substance to be acted upon the albumen present is superficially coagulated; the interior of the mass escaping many times because of a lack of penetrating power in the disinfectant.

Such being the case it has been argued that in the action of bichloride of mercury upon the germs of disease the same effect is produced, *i. e.*, the external gelatinous capsule of the germ is coagulated, while the internal vital portion is left untouched, and if by any means the compound between this covering and the disinfectant be dissolved the micro-organism is quite as ready to go on with its work of destruction as though it had never been acted upon.

Believing this, different experimenters have endeavored to solve the problem by various means, trying one method and then another without definitely proving what they had hoped to do.

One method, which seems very satisfactory so far as showing the effect of certain chemical substances upon the supposed compound formed between the bichloride of mercury and the envelope of the germ, is that used by the author of "Corrosive Sublimate as a Germicide." *

* Medical News, October, 1892.

This method has been used in the experiments given here, and consists of the following: An ordinary test tube is taken, plugged with cotton wool, and through this plug is passed a glass rod containing a plug of the same material for part of its length. In the test tube is a small amount of a non-germicidal alkali, as calcium carbonate, or carbonate of sodium, and sodium chloride. After sterilization the apparatus is ready for use. To a given quantity of the bouillon-culture of the germ used was added a given amount of the $HgCl_2$ strength, 1 to 1000. After a given length of time one c. c. of the disinfected culture was transferred to the prepared test tube and connected with a hydrogen-sulphide generator until sufficient precipitation of the mercury had taken place.

Gelatin, or agar media, was then inoculated with from three to four loops of the preparation and Petri plates made.

The chemical action taking place is very manifest; the hydrochloric acid formed being neutralized by the alkali present, sodium carbonate or calcium carbonate.

The sodium chloride assists in dissolving the compound formed between the bichloride of mercury and the gelatinous material.

The germs used in experimenting were the staphylococcus pyogenes aureus and albus because of their frequent occurrence, and although not spore-forming in the common acceptance of the term, they are nevertheless very resistant to destructive influences.

This work was done to satisfy myself of the efficacy of bichloride of mercury as a germicide under certain conditions, and although the experiments made were comparatively few in number, they were accomplished with all possible care and accuracy.

1893. April 7th. Five c. c. of 1:1000 bichloride of mercury to five c. c. of bouillon-culture of staphylococcus pyogenes aureus, three days old. Time of exposure fifteen, thirty, sixty minutes. Agar plates.

April 11th. The fifteen and thirty minutes plates well developed.

April 27th. Sixty minutes plate still undeveloped.

April 28th. One c. c. of $HgCl_2$ to five c. c. of culture of the staphylococcus pyogenes albus, fifteen days old. Time, twenty-four hours.

Three agar plates made.

May. 8th. All three plates developed.

April 28th. Five c. c. of $HgCl_2$ to five c. c. of culture of the albus, fifteen days old. Time, twenty-four hours. Three agar plates made.

May 8th. No development on any plate.

May 3d. Fifteen c. c. of $HgCl_2$ to five c. c. of culture of the albus, five days old. Time, twenty-four hours. Three gelatin plates made.

May 11th. One colony on each of two plates, the other one being undeveloped.

May 3d. Ten c. c. of $HgCl_2$ to five c. c. of a bouillon culture of the aureus, four days old. Time, twenty-four hours. Three gelatin plates made.

May 11th. No development.

May 9th. Twenty c. c. of $HgCl_2$ to five c. c. of culture of the albus, seven days old. Time, seventy, eighty-five and one hundred minutes and two hours. Two plates for each exposure.

May 18th. No colonies on the plates of the first two exposures. One of the plates having the one hundred minutes exposure exhibited one colony while the other had none. Of the plates having the two hours exposure one plate was undeveloped, while the other possessed three colonies.

May 11th. Five c. c. of $HgCl_2$ to five c. c. of the aureus culture, two days old. Time, fifteen, thirty, forty-five and sixty minutes. Agar plates.

May 22nd. One colony on the plate having the forty-five minutes exposure, none on the other.

May 15th. Two c. c. of $HgCl_2$ to five c. c. of a culture of the albus, seven days old. Time, fifteen, fifty-five and sixty minutes. Gelatin plates.

May 22nd. Plate of the fifty-five minutes exposure very well developed. Others not.

All of these experiments were performed with sodium carbonate used as a neutralizing agent, care being taken that a sufficient quantity was used to serve the purpose. In the succeeding experiments barium carbonate was used instead.

May 16th. Five c. c. of $HgCl_2$ to five c. c. of bouillon-culture of aureus, six days old. Time fifteen, thirty, forty-five and sixty minutes. Gelatin plates.

May 26th. No development in any plate.

May 17th. Two c. c. of $HgCl_2$ to five c. c. of a culture of the albus, seven days old. Time twenty-four hours. Gelatin plates.

May 24th. One colony.

May 23d. Five c. c. of acid solution of $HgCl_2$ 1:1000 to five c. c. of culture of the aureus, two days old. Time, five, ten, fifteen, thirty and sixty minutes. Gelatin plates.

June 6th. No development in any plate.

May 31st. One c. c. of acid solution of $HgCl_2$ to five c. c. of albus, six days old. Time, one, five, ten, fifteen and sixty minutes. Gelatin plates.

June 9th. No development.

June 1st. Three c. c. of acid solution $HgCl_2$ to five c. c. of a culture of the albus, nine days old. Time, one, five, fifteen and thirty minutes. Gelatin plates.

June 9th. No development.

A little experimenting was done with a solution of $HgI_2$ 1:-500 to test its germicidal powers.

When precipitation of the mercury by hydrogen sulphide occurred the solution was found to be valueless as a germicide, while without the action of the hydrogen sulphide its effect upon the germs was immediate.

May 19th. One c. c. of $HgI_2$ 1:500 to five c. c. of the albus culture, four days old. Time, one, five, ten, and fiften minutes. [$H_2S$ employed].

May 22d. All profusely developed excepting the plate of the ten minutes exposure, which had no colonies.

May 19th. One c. c. $HgI_2$ to five c. c. of the aureus culture, three days old. Gelatin plates. Time, one, five, ten and fifteen minutes, without precipitation by $H_2S$.

May 31st. No development in any plate.

May 23d. Five c. c. of $HgI_2$ to five c. c. of a culture of the albus, four days old. [Precipitation of mercury by $H_2S$]. Time, thirty minutes. Gelatin.

May 31st. Very well developed.

In looking over the results presented here one will notice now and again a seeming irregularity in the development of a plate. For instance: A plate of fifteen minutes exposure may exhibit no colonies while one of sixty minutes, of the same series, may be well developed. This may be explained by the supposition that only the more feebly resistant germs were transferred to the gelatin in the first case, and being destroyed by the germicide before the transference no development could consequently take place. In the latter case a sufficient number of the more resistant germs were brought over to show development under suitable conditions.

Again, enough of the germicide may have been taken over with the germs to have formed a sterilized area around the micro-organisms preventing their growth in the one case, while in the other the disinfectant was sufficiently distributed throughout the gelatin to prevent any thing of the kind. Care was taken to accomplish this in every case, however.

The neutralization of the acid formed was also especially looked after.

In comparing the results obtained from the use of the ordinary solution of bichloride of mercury, and that of the acid solution, it will be noticed that the latter is far more prompt and efficient in its action than the former, due probably to the excess of hydrochloric acid in the acidulated solution.

Although it is manifestly evident that bichloride of mercury is not sufficiently penetrating in its action to serve as a germicide, excepting under conditions most favorable to its complete performance of the results desired, yet the ideal germicide has not been discovered, and those that are so efficient as bichloride of mercury are hard to find.

It would seem from the experiments of others that the more concentrated solutions of this disinfectant are still less to be relied upon in their action than the strengths more ordinarily used, from the fact that the combination between the bichloride of mercury and the capsule of the germ is more quickly effected, and although the portion influenced is probably of a firmer consistency than that brought about by using a solution of weaker strength, still, the amount of penetration is not so great, and the desired results not so nearly accomplished.

Because of its extremely poisonous nature bichloride of mercury will never be extensively used alone in dentistry, yet with a proper knowledge of its powers and those of other germicidal agents of a high degree of usefulness, a combination may be effected in the future which may prove of the utmost value to the oral surgeon of the coming times.

